# Biochemical and biophysical characterization of pathological aggregation of amyloid proteins

**DOI:** 10.52601/bpr.2022.210032

**Published:** 2022-02-28

**Authors:** Houfang Long, Shuyi Zeng, Yunpeng Sun, Cong Liu

**Affiliations:** 1 Interdisciplinary Research Center on Biology and Chemistry, Shanghai Institute of Organic Chemistry, Chinese Academy of Sciences, Shanghai 201210, China; 2 University of Chinese Academy of Sciences, Beijing 100049, China; 3 Bio-X Institutes, Key Laboratory for the Genetics of Developmental and Neuropsychiatric Disorders, Ministry of Education, Shanghai Jiao Tong University, Shanghai 200030, China; 4 Bio-X-Renji Hospital Research Center, Renji Hospital, School of Medicine, Shanghai Jiao Tong University, Shanghai 200240, China

**Keywords:** Amyloid protein, Pathological fibrillation, Transmission electron microscopy, α-Synuclein, Aggregation kinetics, Neurodegenerative diseases, Parkinson’s disease

## Abstract

Protein amyloid fibrillation, a process of liquid to solid phase transition, is involved in the pathogenesis of a variety of human diseases. Several amyloid proteins including α-synuclein (α-syn), Tau, amyloid β (Aβ) protein, and TAR DNA-binding protein 43 kDa (TDP-43) form pathological fibrils and deposit in patient brains of different neurodegenerative diseases (NDs) such as Parkinson’s disease (PD), Alzheimer’s disease (AD) and Amyotrophic lateral sclerosis (ALS). Preparation and characterization of amyloid fibrils *in vitro* are essential for studying the molecular mechanism underlying the dynamic amyloid aggregation and its pathogenesis in diseases. In this protocol, we take PD-associated α-syn as an example, and describe amyloid protein purification and fibrillation approaches. We then introduce biochemical and biophysical characterization of amyloid fibrils by Thioflavin-T (ThT) fluorescence kinetics assay, transmission electron microscopy (TEM), atomic force microscopy (AFM) and multiple fibril stability measurement assays. The approaches described here are applicable to different amyloid proteins, and are of importance for further study on the structure determination of amyloid fibrils and their pathological function in cells and animal models.

## INTRODUCTION

Pathological fibrils formed by different amyloid proteins are widely observed and commonly regarded as histopathological hallmarks of different neurodegenerative diseases (NDs), including α-synuclein (α-syn) in Parkinson’s disease (PD) (Baba* et al.* 1998; Spillantini* et al.* 1997), Tau and amyloid β (Aβ) in Alzheimer’s disease (AD) (Goedert* et al.* 1988; Murphy and LeVine 2010), TAR DNA-binding protein 43 kDa (TDP-43) and fused in sarcoma (FUS) in amyotrophic lateral sclerosis (ALS) (Arai* et al.* 2006). Mounting evidence demonstrates that amyloid fibrils exhibit a wide range of different pathological properties including induction of protein homeostasis collapse (Hipp* et al.* 2019), propagation and spread among different cells (Luk* et al.* 2012; Mao* et al.* 2016), sequestration of mitochondria and endoplasmic reticulum (ER) (Forno and Norville 1976; Shahmoradian* et al.* 2019; Watanabe* et al.* 1977), disruption of cell membrane (Lashuel* et al.* 2002; Tsigelny* et al.* 2012; Volles and Lansbury 2002), and so on. Thus, pathological amyloid fibrils play an essential role in disease initiation and progression. The preparation and systematic characterization of different amyloid fibrils are important for understanding how different proteins undergo a liquid–solid phase transition and dynamically assemble into insoluble fibrils, which may provide a basis for investigating the pathological activity of amyloid fibrils in NDs. In addition, assays measuring chemical and physical properties of fibrils will help not only for investigating the relationship of fibril structure and cytotoxicity but also for the development of stabilizer or destabilizer of amyloid fibrils for therapeutic application.

### Overview of the protocol

We summarize and provide a systematic protocol, which is mainly composed of five parts, including: (1) purification of amyloid protein; (2) preparation of amyloid fibril and pre-formed fibrils (PFFs); (3) morphological characterization of amyloid fibrils using transmission electron microscopy (TEM) and atomic force microscopy (AFM); (4) measurement of aggregation kinetics; (5) examination of fibril stability. α-Syn is taken as an example. α-Syn contains 140 amino acids and is an intrinsically disordered protein in solution which can form amyloid fibrils *in vitro* or under disease conditions (Baba* et al.* 1998; Chen* et al.* 1995; Guerrero-Ferreira* et al.* 2018; Li* et al.* 2018; Spillantini* et al.* 1997; Tu* et al.* 1998). The paper aims to establish a protocol for the preparation of α-syn fibrils and the systemic characterization of α-syn fibrils formed *in vitro*. Overall, we firstly reconstruct and purify full-length wild-type (WT) α-syn protein. Then α-syn fibrils are produced and further sonicated into PFFs. Next, TEM and AFM are used to examine the morphology of α-syn fibrils including the pitch, width and helicity of fibrils. The nucleation-dependent fibrillation of α-syn is monitored by ThT kinetics assay *in vitro*. For further assessing the chemical and physical stability of α-syn fibrils, different approaches are performed including proteolytic digestion with proteinase K (PK), sonication assay and freeze-thaw assay.

### Applications and advantages of the protocol

The protocol covers several major steps for amyloid fibril preparation and characterization *in vitro*, which starts with the amyloid protein sample purification and fibril preparation, and ends up with a wide range of biophysical and biochemical approaches to systematically characterize the kinetics and morphology of amyloid fibril from different aspects. In this protocol, α-syn is used as an example protein for detailed approach description. These approaches can be applied to other amyloid proteins such as Tau, FUS, and TDP-43 for their fibril preparation, fibrillation kinetics assessment, as well as biophysical and biochemical characterization. This protocol is not only useful for the mechanistic study of pathological fibrils by biochemists and structural biologists, but also cell biologists and pathologists in their study of fibril pathology in cell and disease-related animal models.

### Limitations of the protocol

( 1 ) The protein purification method described in the protocol is optimized for α-syn protein. As for other amyloid proteins, the detailed purification assay may need to modify (*e*.*g*., using different purification columns and buffers) depending on their own biochemical properties.

( 2 ) For the characterization of fibril morphology using AFM, different types of fibrils may feature distinct surface properties, which in turn may influence the adsorption efficiency of fibrils on mica flakes. Therefore, it is necessary to conduct a comprehensive analysis of AFM data combined with the results from TEM.

( 3 ) For ThT assay, amyloid fibrils with different structures may exhibit distinct binding capability and binding patterns to ThT. Thus, the intensity of ThT fluorescence signal from different amyloid fibrils cannot be used to assess and compare the fibril yield among different fibrils. Alternatively, the combination of TEM and SDS-PAGE gel can be used to evaluate the fibril yield.

## SUMMARIZED PROCEDURE

### α-Syn protein purification

( 1 ) Culture bacteria that overexpress α-syn protein with 2× YT medium (liquid microbial growth medium).

( 2 ) Induce expression of α-syn with 1 mmol/L isopropyl-1-thio-D-galactopyranoside (IPTG).

( 3 ) Lyse bacteria with a high-pressure crusher after harvest.

( 4 ) Multiple steps for lysate treatments including boiling, the addition of streptomycin sulfate, adjusting pH of the supernatant to 3.5, followed by dialysis overnight.

( 5 ) Purify lysate using anion exchange and Superdex 75 columns.

( 6 ) Verify the purity of protein and store aliquots at –80 °C.

### α-Syn fibrillation and PFFs preparation

( 1 ) Incubate α-syn monomer in fibril growth buffer in ThermoMixer.

( 2 ) Prepare α-syn PFFs with a sonicator.

### Morphology characterization of α-syn fibril

( 1 ) Deposit fibrils on a glow discharged grid with carbon film. Images of α-syn fibrils and PFFs are captured by Tecnai T12 microscope.

( 2 ) Deposit fibrils on the surface of mica. Use AFM to image fibrils and measure the width, pitch and helicity of fibrils.

### Measurement of aggregation kinetics with ThT assay

( 1 ) Mix α-syn monomer, α-syn PFFs and ThT into fibril growth buffer.

( 2 ) Place samples in a 384-well optical plate in triplicate and monitor by a Fluoroskan Ascent microplate reader.

( 3 ) Characterize the fibrils formed in the ThT assay using TEM.

### Proteolytic digestion of α-syn PFFs

( 1 ) Calibrate the concentration of α-syn fibrils and sonicate fibrils into PFFs.

( 2 ) Incubate α-syn PFFs with PK at 37 °C for different digestion duration.

( 3 ) Stop the digestion reaction by adding phenylmethylsulfonyl fluoride (PMSF) and assess the fibril samples using SDS-PAGE gel.

### Physical stability measurement of α-syn fibrils by sonication and freeze-thaw assay

( 1 ) Fragment fibrils with different sonication duration.

( 2 ) Characterize the size of PFFs using TEM.

( 3 ) Rapidly freeze and thaw fibrils for multiple cycles.

( 4 ) Monitor the secondary structure of fibrils by using circular dichroism (CD) spectrometer.

## PROCEDURE

### α-Syn protein purification [TIMING 2 d]

( 1 ) First transfer glycerol cryopreserved bacteria which overexpress human WT α-syn to 20-mL 2× YT medium at 37 °C, 220 r/min, overnight.

( 2 ) Then inoculate 10-mL bacterial liquid into 1-L 2× YT medium to allow bacteria to grow at 37 °C, 220 r/min, 5 h.

( 3 ) Induce α-syn protein expression with IPTG at a final concentration of 1 mmol/L at 37 °C, 220 r/min, 4 h.

( 4 ) Harvest bacteria by centrifugation (4,500 r/min, 4 °C, 20 min) and resuspend them with bacteria storage buffer.

( 5 ) Lyse the bacteria with a high-pressure crusher and add protease inhibitor, 1 mmol/L PMSF, at 4 °C, 10 min.

**[TIP]** Crush bacteria solution to clear and translucent. Avoid air intake during the process.

( 6 ) Boil the lysate for 15 min to remove impurities.

( 7 ) To remove nucleic acids (Liang* et al.* 2009), add streptomycin sulfate powder to the supernatant fraction (20 mg/mL, *w*/*v*) after centrifugation (15,000 r/min, 4 °C, 20 min), at 4 °C, 30 min, stirring.

( 8 ) Adjust the pH of the supernatant to 3.5 with 2 mol/L hydrochloric acid (HCl) after centrifugation (15,000 r/min, 4 °C, 20 min).

**[TIP]** Wear masks, gloves and other protective equipment to prevent hydrochloric acid from harming the human body.

( 9 ) Dialyze lysis solution in Buffer A at 4 °C, overnight.

(10) Load the protein solution onto the Q column at a speed of 2 mL/min after equilibrated with Buffer A, followed by Buffer A to rinse the unbounded protein. Then elute the target protein with 0–60% Buffer B within 40 min.

**[TIP]** Before loading, the protein solution is filtered with a 0.22-µm filter. And be sure to rinse the unbounded protein completely before elution.

(11) Collect the target peak of Q column spectrum, and load the fraction onto Superdex 75 chromatography after concentration.

(12) Collect α-syn protein and verify the purity of protein with SDS-PAGE gel. α-Syn protein after concentration is divided into aliquots and stored at –80 °C, avoiding repeated freeze-thaw cycles.

**[TIP]** Protein concentration should not be too high to avoid protein precipitation. The appropriate concentration is 100–500 μmol/L.

### α-Syn fibrillation and PFFs preparation [TIMING 8~12 d]

(13) Incubate α-syn protein (200 µmol/L, 200 µL) in fibril growth buffer with ThermoMixer, at 37 °C with constant agitation (900 r/min) for 5–7 d.

(14) Characterize the morphology of mature α-syn fibril using TEM.

(15) Fragment α-syn fibrils into PFFs by JY92-IIN sonicator, with 20% power, 1 s on, 1 s off, 30 s. Characterize the morphology of PFFs using TEM.

**[TIP]** Be careful to avoid bubble formation.

(16) For the second cycle of α-syn fibrillation, incubate α-syn WT monomer in the presence of PFFs (0.5 mol%), which were prepared in Step 15, with ThermoMixer (37 °C, 900 r/min, 3–5 d).

**[TIP]** α-Syn fibrils/PFFs are divided into aliquots and stored at –80 °C, avoiding repeated freeze-thaw cycles.

### Morphology characterization of α-syn fibril/PFFs with TEM [TIMING 30 min]

(17) Place copper grids (200 mesh) with coated carbon support film facing up on a parafilm-covered slide. Transfer the slide to the vacuum chamber of the glow discharge cleaning system.

**[TIP]** Keep the front side of grids up. Pick up grids from their edge with tweezers carefully to avoid bending the grids and damaging carbon layers.

(18) Set glow discharge time for 45 s and vacuum pressure at 0.26 mBar to achieve ideal plasma intensity. A violet plasma will be observed and then the grids glow discharged.

**[TIP]** Charges on the glow discharged grids just stay for only 2 h. Re-glow discharge grids if the interval is too long.

(19) Dilute α-syn fibrils and PFFs to 20 μmol/L with fibril growth buffer.

**[TIP]** Ensure that α-syn fibrils and PFFs are evenly distributed in the solution by vortex and pipetting up and down before dilution.

(20) Place a parafilm (10-cm long) on a bench. Then pipette a drop of 5 μL deionized water and two drops of 5 μL 3% (*w*/*v*) uranyl acetate for per grid on the parafilm.

(21) Hold the glow discharged grids with Dumont N4 negative-action medical and biological tweezers and leave grids suspended in midair.

(22) Load 5 μL of α-syn fibrils or PFFs sample (20 μmol/L) onto the carbon film side of grids and incubate for 45 s.

(23) Remove sample solution on the grid by gently blotting the grid with filter paper.

(24) Wash the grid with 5 μL deionized water on the parafilm by gently touching the grid with the drop.

(25) Wash the grid with 5 μL 3% (*w*/*v*) uranyl acetate and remove the uranyl acetate on the grid by filter paper.

(26) Adsorb another drop of 5 μL 3% (*w*/*v*) uranyl acetate on the grid, and incubate for 45 s.

(27) Remove uranyl acetate on the grid by blotting grids with the edge of filter paper gently.

(28) Leave grids dry in the air and store them in a labeled box.

(29) Images are captured by Tecnai T12 microscope.

### Morphology characterization of α-syn fibril with AFM [TIMING 30 min]

(30) Attach the mica to the AFM metal specimen discs (12 mm) with double-coated adhesive tape.

(31) Adhere the mica layers with tape, and then separate them in a quick motion. After removing the top layer of mica, the newly cleaved and clean mica surface is ready for use.

**[TIP]** Flatten the adhesive tap onto the surface of mica to remove the bubbles between these two surfaces. Several attempts may need to be taken to ensure the surface of the mica is flat.

(32) Load a drop of 5 μL α-syn fibrils or PFFs sample (20 μmol/L) onto the surface of mica and incubate for 3 min at RT.

(33) Rinse the surface of mica with deionized water to remove unbound fibrils.

(34) Leave the surface of mica dry with nitrogen flow.

(35) The mica surface is probed in the air by Multimode 8 scanning probe microscope on scanAsyst mode. Images are processed with NanoScope Analysis 1.5 software (Bruker).

### Measurement of aggregation kinetics with ThT assay [TIMING 3–5 d]

(36) Dilute α-syn monomer to 50 μmol/L with fibril growth buffer, and distribute it into 1.5-mL tubes, 160 μL per tube.

**[TIP]** Ensure that the plate, buffer and reagents used are clean. Contaminations may easily influence the protein aggregation kinetics.

(37) Add 0.8 μL PFFs with a concentration of 50 μmol/L (0.5 mol%, relative to α-syn monomer) to α-syn monomer solution.

(38) Add ThT with a final concentration of 10 μmol/L to the reaction system.

**[TIP]** Mix the solution in the tube thoroughly.

(39) Dispense samples into a 384-well optical plate, 50 μL/well, in triplicate.

**[TIP]** Avoid air bubbles.

(40) Monitor the fluorescent signal with a Fluoroskan Ascent microplate reader with 440 nm excitation light and 485 nm emission light, bottom reading, 900 r/min, at 37 °C, about 85 h.

(41) Characterize the ThT samples at the endpoint using TEM.

**[TIP]** Pay attention to fully mixing before sampling to prevent sample deposition.

(42) Concentrate α-syn fibrils formed in the ThT assay by centrifugation (14,462 *g*, 25 °C, 45 min) to separate the soluble monomer (supernatant, S) and insoluble fibril (pellet, P).

(43) Dissolve the pellet fraction with insoluble buffer (Mao* et al.* 2016) and boil it for 30 min.

(44) Boil the supernatant and dissolved pellet solution for 15 min after adding loading buffer.

(45) Load samples on SDS-PAGE gel and stain gel with Coomassie brilliant blue.

(46) Capture and analyze images with Image Lab 3.0 (Bio-Rad).

### Proteolytic digestion of α-syn PFFs [TIMING 4 h]

(47) Concentrate α-syn fibril by centrifugation (14,462 *g*, 25 °C, 45 min) to remove the residual monomer. Record the volume of supernatant (*V*_s_) and test the concentration of residual soluble α-syn (*C*_s_) with Nanodrop.

**[TIP]** Do not pipet the pellet fraction when removing the supernatant.

(48) Calibrate the concentration of α-syn fibril by subtracting the amount of residual soluble α-syn with the total amount of α-syn monomer (*V*_t_ × *C*_t_), shown as *C*_p _= (*V*_t_ × *C*_t _− *V*_s_ × *C*_s_) / (*V*_t _− *V*_s_). And resuspend the fibril pellet with phosphate buffer saline (PBS, pH 7.4) to 50 μmol/L.

(49) Produce α-syn PFFs by sonication as described above. Incubate PFFs (50 μmol/L) with 0.5 μg/mL PK at 37 °C for 0, 10, 30, 60, 120 min, separately.

**[TIP]** Different concentrations of PK may be tested for digesting different types of fibrils.

(50) Add PMSF with a final concentration of 1 mmol/L to stop the digestion.

(51) Boil samples with SDS-loading buffer at 100 °C for 15 min and load samples on 4%–20% Bis-Tris gels, then stained by Coomassie brilliant blue.

(52) Capture and analyze images with Image Lab 3.0 (Bio-Rad).

### Physical stability measurement of α-syn fibrils by sonication and freeze-thaw assay

#### *Sonication assay* [*TIMING 2 h*]

(53) Dilute α-syn fibrils with fibril growth buffer to 50 µmol/L.

(54) Produce α-syn PFFs by sonication at 20% power, *n*× (1 s on, 1 s off), *n* = 2, 15.

**[TIP]** The “*n*” can be tested between “0–15” times for different fibril samples.

(55) Characterize the distribution of the length of PFFs using TEM.

#### *Freeze-thaw assay* [*TIMING 2h*]

(56) Freeze α-syn fibrils (20 µmol/L) quickly in liquid nitrogen and warm them up in the water bath, as one freeze-thaw cycle.

**[TIP]** The number of freeze-thaw cycles can be optimized for different fibrils.

(57) Measure the secondary structure of fibrils with CD, which is recorded from 200 to 260 nm.

(58) Analyze the data with Pro-Data Viewer (version 4.4.2.0).

**[?TROUBLESHOOTING]**

Troubleshooting tips are listed in Table 1.

**Table 1:** Troubleshooting table

Step	Problem observed	Possible reason	Solutions
12	Failure of purification of pure α-syn protein	(1) Protein induced expression failure (2) Inaccurate elution of salt concentration of Buffer B used in Step 10	(1) Confirm the successful expression of the protein before purification (2) Confirm the accuracy of buffer preparation
14	α-Syn fibrils bundle together from TEM imaging	The concentration of fibril sample used for TEM is too high	Dilute the fibril sample to 10–50 µmol/L
15	The length of PFFs is too long or uneven	Bubble forms during sonication	Avoid bubble formation
16	α-Syn fibril or PFFs are depolymerized	Too long storage time or repeated freeze-thaw	Avoid long-term storage and avoid repeated freeze-thaw
18	Grids exhibit low sample adsorption capacity	(1) Low glow-discharge quality of grids (2) Effect of fibril solution	(1) Re-glow discharge grids (2) Optimize fibril solution
31	The AFM probe is easily damaged	Rough surface of mica	Make sure that the mica has a flat surface
36	Fluorescence signal is abnormal	Contamination of the plate, buffer or reagents	Try to use a clean plate, fresh buffer and new reagents
39	Results are of poor reproducibility	Improper operation or too few sample repeat	Set up more sample repeats
41	The amount of fibril produced doesn’t match the ThT result	Sample deposition during sampling	Mix well before sampling
45	The pellet sample is stuck on the top of gel	Incomplete dissolution of the fibril pellet	Sonicate the pellet solution or boil it for over 30 min
48	The yield of fibrils is low	(1) Protein degradation (2) Too low concentration of monomer for incubation	(1) Confirm the quality of protein (2) Increase the concentration of monomer to 200–500 µmol/L
54	There is no significant difference between two fibril strains after sonication	Inappropriate sonication time (*n*)	Try different sonication time (*n*)

## ANTICIPATED RESULTS

### Purification of monomeric α-syn protein

**Figure 1:**
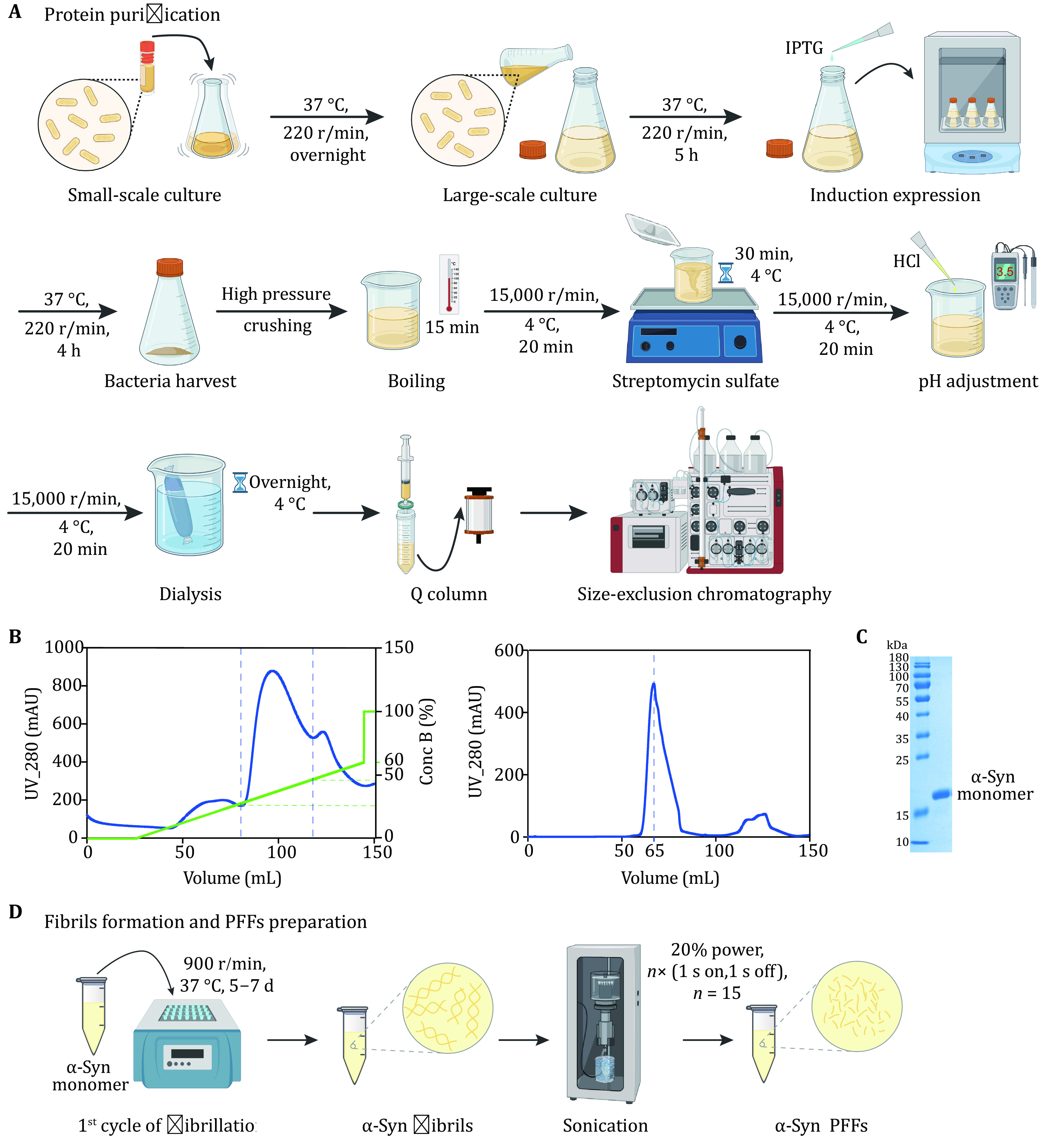
Expression and purification of α-syn. **A** The schematic diagram of α-syn expression and purification. Anion exchange chromatography and size exclusion chromatography are applied to protein purification. **B** The anion-exchange (left) and size exclusion (right) chromatographic purification of α-syn. α-Syn is collected when the concentration of Buffer B (green) is 20%–40% during ion exchange chromatography. **C** The result of SDS-PAGE after purification. The marker of protein (kDa) is labeled on the left of gel. **D** The schematic diagram of α-syn fibril and PFFs preparation

Bacteria overexpressing α-syn protein were grown and IPTG at a final concentration of 1 mmol/L was used to induce the expression of α-syn protein. Then bacteria were harvested and crushed to purify the protein. Bacteria lysate was boiled to remove impurities and treated with streptomycin sulfate to remove nucleic acid. In addition, the pH of lysate was adjusted to 3.5 and the lysis solution was on dialysis overnight at 4 °C (Fig. 1A). To purify α-syn protein, the solution was loaded on the anion exchange column and eluted with 0–60% Buffer B in 40 min (Fig. 1A). The solution of the peak at 20%–40% Conc B contained α-syn protein (Fig. 1B) and the fraction was further concentrated to load Superdex 75 chromatography (Fig. 1A). And the peak of α-syn monomer appeared after eluting about 60 mL buffer (Fig. 1B). To confirm the fraction, the extracts were performed Coomassie Brilliant Blue assay and the gel image was acquired. The result confirmed monomeric α-syn protein with high purity (Fig. 1C).

### α-Syn fibrillation and morphology characterization

**Figure 2:**
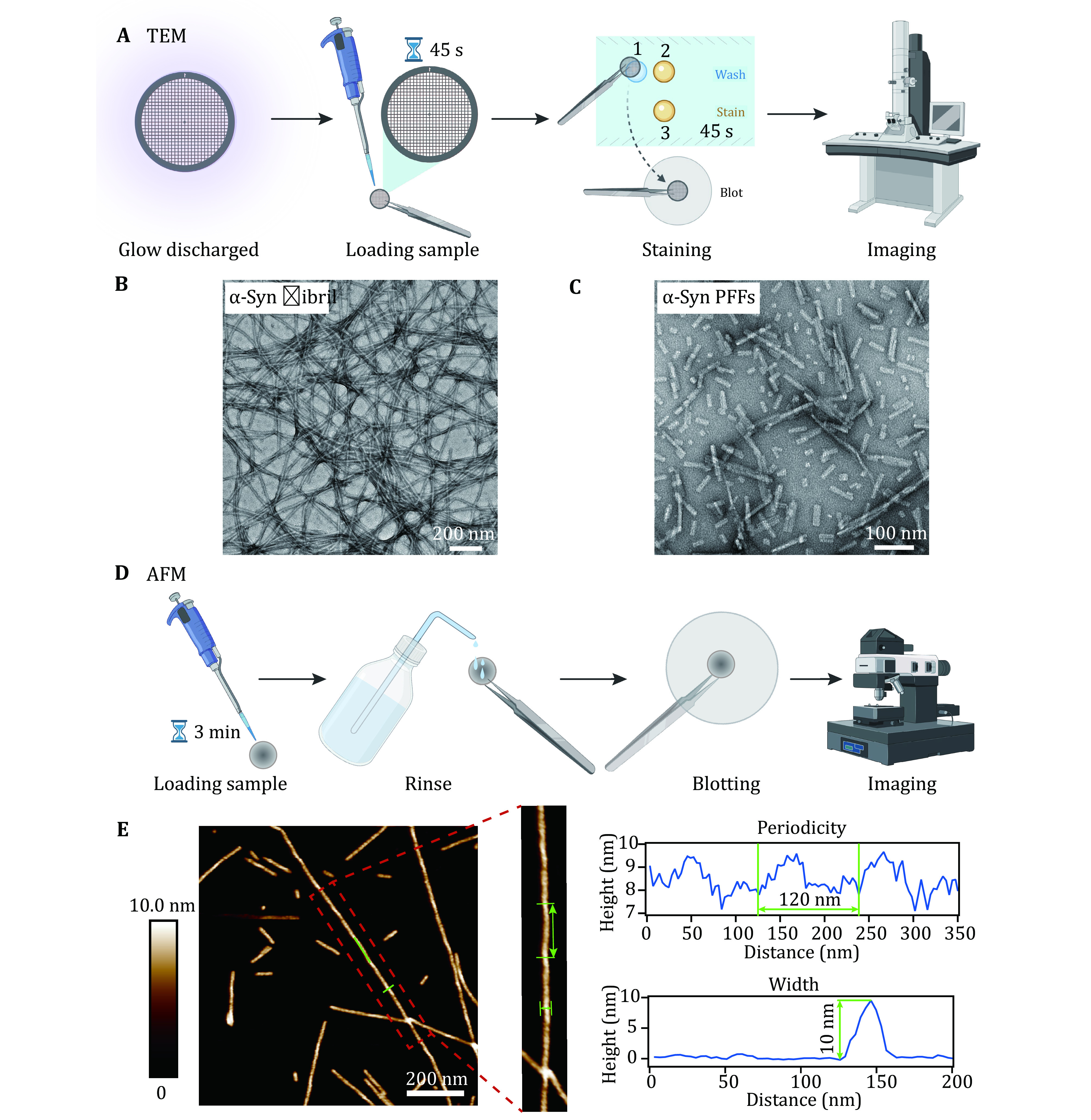
Characterization of α-syn fibril and PFFs by TEM and AFM. **A** Sample preparation for TEM imaging. **B**, **C** Negative-staining TEM images of α-syn fibrils (B) and α-syn PFFs (C), scale bar, 200 nm. **D** Sample preparation for AFM imaging. **E** Characterization of the helical parameters of the α-syn fibril by AFM. 2D AFM image is shown, scale bar, 200 nm. Analyses of the cross section and along the fibril are indicated with blue lines and highlighted with green lines

For the first cycle of fibrillation, α-syn protein in fibril growth buffer was incubated in the ThermoMixer, at 37 °C with constant agitation for 5−7 d (Fig. 1D). The α-syn fibril and PFF samples were characterized by TEM and AFM. For the preparation of TEM imaging, the sample (20 μmol/L, 5 μL) was loaded on the glow discharged grid for 45 s. Then the grid was rinsed with deionized water and uranyl acetate once in turn, and stained with uranyl acetate for 45 s (Fig. 2A). The image was captured by Tecnai T12 microscope. As shown in Fig. 2B, homogeneous and unbranched fibrils were formed. And the α-syn PFFs produced by sonication displayed similar fibril length and fibril morphology (Fig. 2C). α-Syn fibrils were also loaded on a mica and imaged by AFM (Fig. 2D). From the image of AFM, we further confirmed that the half pitch of the fibril was ~120 nm and the width was ~10 nm (Fig. 2E).

### Measurement of aggregation kinetics with ThT assay

**Figure 3:**
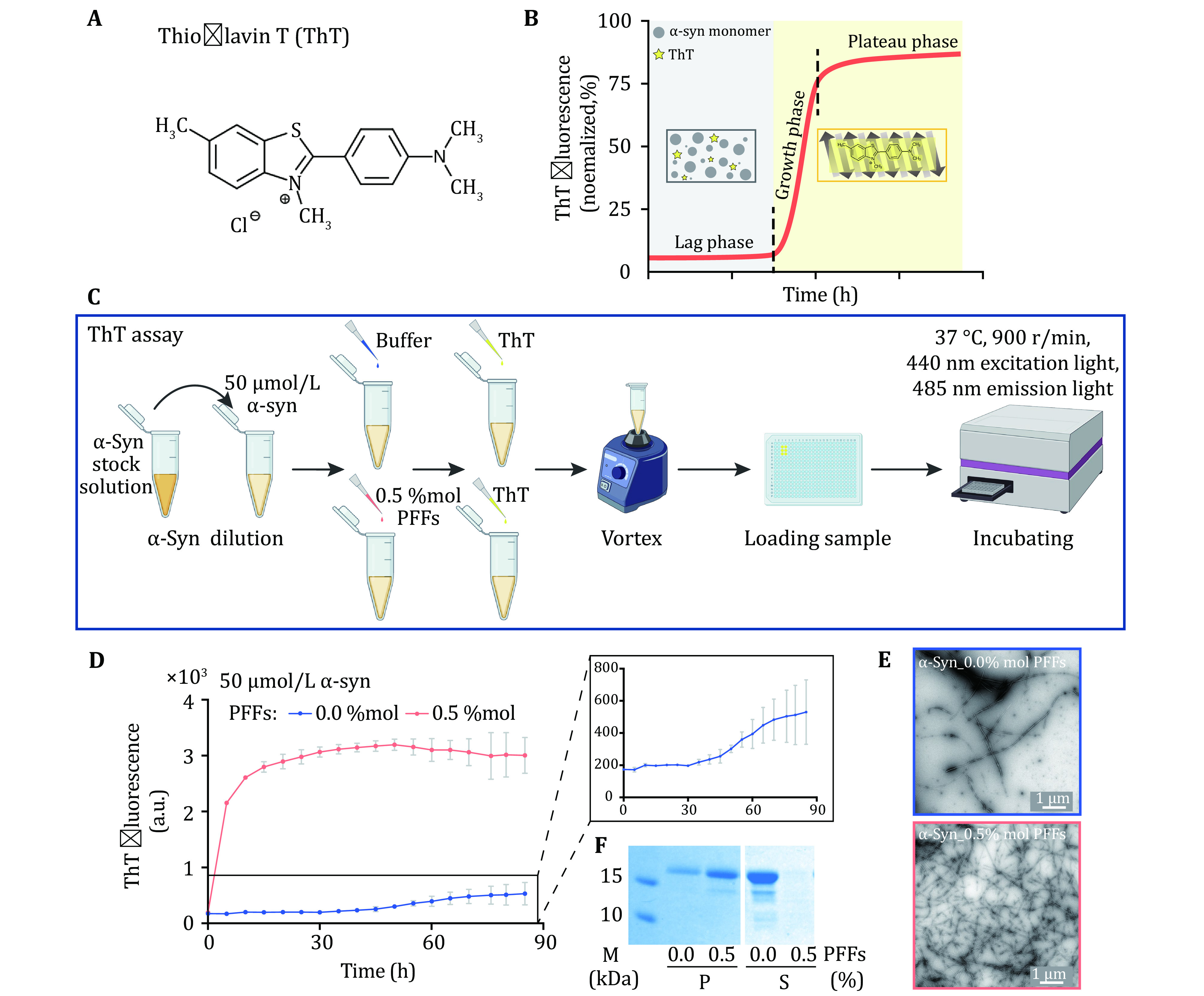
ThT kinetics assay of α-syn fibrillation. **A** The structural formula of ThT molecule. ThT binds to the β-sheet structure of amyloid fibril which results in florescence emission at 485 nm. **B** As amyloid protein aggregates into fibrils, the fluorescence signals increase as well. The aggregation process can be divided into three phases including the lag phase, the growth phase and the plateau phase. In the initial lag phase, amyloid protein samples may self-assemble into different amyloid oligomers without forming fibrils. Then, monomers aggregate into fibrils quickly during the growth phase. Fibril formation is completed during the plateau phase. **C** The schematic diagram of ThT assay. α-Syn PFFs act as the seed to trigger the protein aggregation. **D** ThT kinetics assay for α-syn monomer seeded (red) or not seeded (blue) by the PFFs. Notice that, in the presence of PFFs as seeds, α-syn monomer directly enters the growth phase for fibrillation. The concentration of PFFs is indicated. The aggregation curve of α-syn monomer is magnified. Data shown are mean ± SD, *n* = 3 individual independent samples. **E** The samples collected at the end of the ThT assay were imaged by TEM, scale bar: 1 µm. **F** The pellet (P) fraction (left) and supernatant (S) fraction (right) of ThT samples after centrifugation are loaded on SDS-PAGE gel, respectively

ThT is a benzothiazole dye that exhibits enhanced fluorescence upon binding to amyloid fibrils (Fig. 3A). Fibril formation mainly goes through three stages, including lag phase (nucleation), growth phase (elongation) and plateau phase (saturation) (Fig. 3B). Once the β-sheet structure is formed, the ThT dye binds to it to generate a fluorescent signal, which is used to monitor the formation of fibril (Fig. 3B). α-Syn protein at a final concentration of 50 μmol/L in fibril growth buffer which was added with 0.5% mol PFFs or buffer was incubated in a microplate reader (Fig. 3C). The fluorescence signal of α-syn seeded by PFFs quickly reached a plateau in 15 h. Whereas, the accumulation of α-syn itself required a lag time of about 30 h (Fig. 3D). And the formation and yeild of fibrils can be detected by TEM (Fig. 3E) and SDS-PAGE gel (Fig. 3F). The result suggested that α-syn PFFs effectively seeded monomeric α-syn protein *in vitro* and the kinetics of fibril formation can be monitored by ThT assay.

### Measurement of chemical and physical stability of α-syn fibrils

**Figure 4:**
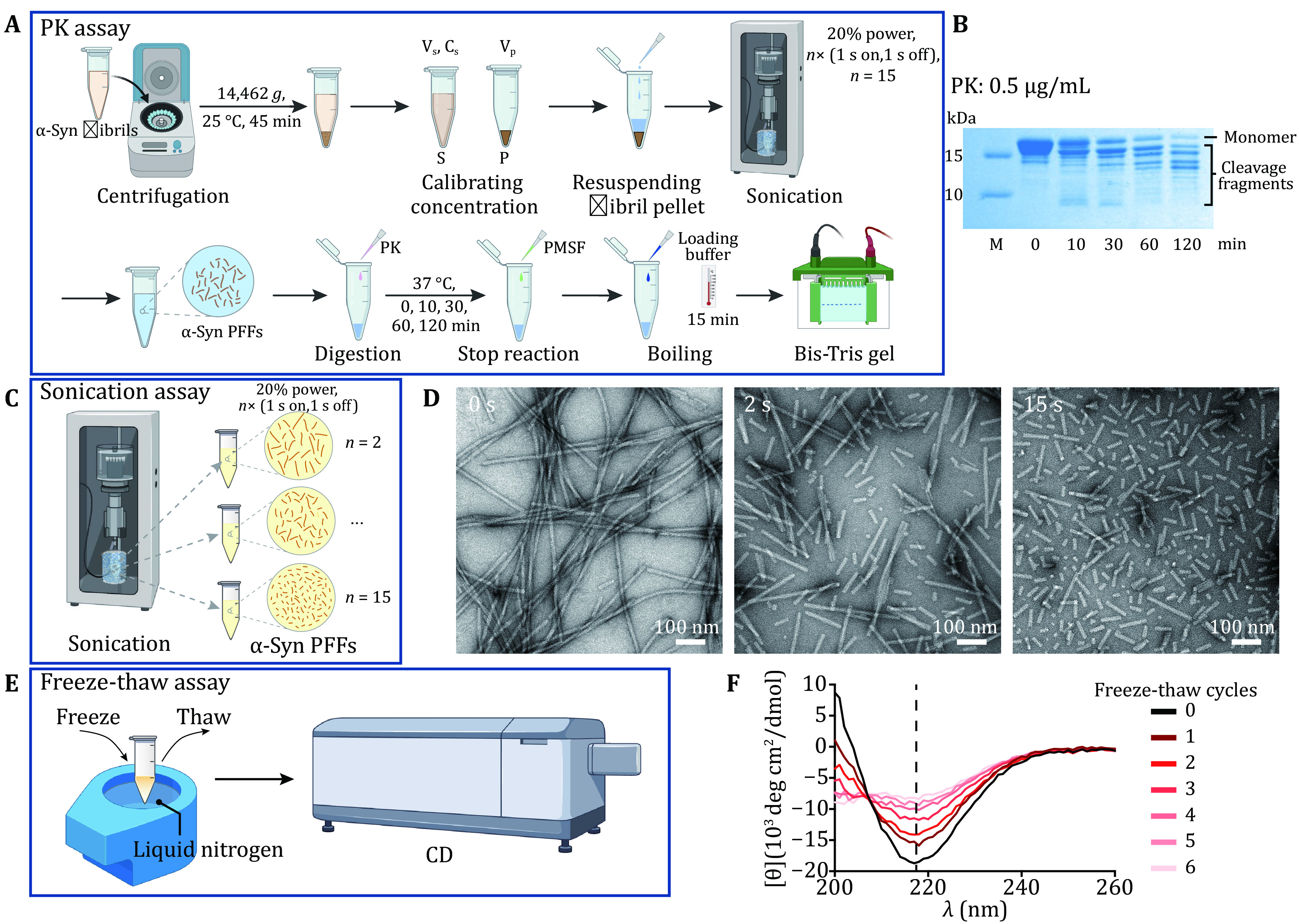
Characterization chemical and physical stability of α-syn PFFs. **A** The schematic diagram of PK digestion assay. S, supernatant; P, pellet; V_s_, volume of supernatant; C_s_, concentration of supernatant; V_p_, volume of pellet. **B** Proteolytic digestion of α-syn PFFs at several time points, including 0, 10, 30, 60 and 120 min. **C** The schematic diagram of sonication assay. **D** Representative TEM images of α-syn PFFs by sonicated for *n*× (1 s on, 1 s off), *n* = 0, 2, 15, scale bar: 100 nm. **E**, **F** The schematic diagram (E) and CD spectra (F) of the freeze-thaw assay. The CD signal at 218 nm which represents the β-sheet content is measured and compared between different cycles. The number of freeze-thaw cycles is marked on the right side of the graph. Note, the fibril tested by CD is one α-syn mutant fibril strain

To examine the chemical stability of α-syn fibrils, we performed PK assay described in Fig. 4A. We calibrated the concentration of α-syn fibrils before sonication. Then α-syn PFFs were digested with PK for 0, 10, 30, 60, 120 min at 37 °C, separately (Fig. 4A). The result showed that PFFs were gradually degraded into fragmentations as time went by (Fig. 4B). In addition, α-syn fibril was treated by different sonication durations (*e*.*g*., 2 or 15 times) (Fig. 4C). Then α-syn PFFs were examined by TEM. As Fig. 4D shows, PFFs processed by different sonication durations had different lengths. What’s more, when α-syn fibrils were quickly frozen and thawed for some cycles (Fig. 4E), the CD spectra showed that fibrils gradually lost the β-sheet structure which represented the fibril amount as the number of freeze-thaw cycles increased (Fig. 4F). These results demonstrated that the stability of fibrils can be detected by these three assays.

## MATERIALS

### Biological materials

• BL21 (DE3) (TransGen Biotech, cat. no. CD601-02)

### Reagents

• Tryptone (Oxoid, cat. no. LP0042)

• Yeast extract (Oxoid, cat. no. LP0021)

• Bis-Tris (Sangon Biotech, cat. no. A610079-0100)

• EDTA (Sangon Biotech, cat. no. A100105-0500)

• Thioflavin T (Sigma Aldrich, cat. no. T3516)

• Proteinase K (Invitrogen, cat. no. 25530031)

• Uranyl acetate

• NaN_3_ (Sigma Aldrich, cat. no. S2002)

• NaCl (Sangon Biotech, cat. no. A100241-0500)

• KCl (Sangon Biotech, cat. no. A501159-0500)

• HCl (Hushi, cat. no. 10011018)

**[CAUTION!]** HCl is highly toxic, corrosive, unstable and explosive. Avoid flames and handle in a fume hood. Be sure to wear lab-gowns, masks and gloves when operating. Dispose should be in accordance with local regulations.

• PBS (Sangon Biotech, cat. no. E607008-0500)

• Tris (Sangon Biotech, cat. no. A501492-0500)

• IPTG (Sangon Biotech, cat. no. A600168-0100)

• SDS (Sangon Biotech, cat. no. A600485-0500)

• Glycerol (Hushi, cat. no. 10010618)

• Streptomycin sulfate (A610494-0250)

• PMSF (Sangon Biotech, cat. no. A610425-0025)

**[CAUTION!]** PMSF is toxic. Handle with protective gloves.

• Isopropanol (Hushi, cat. no. 80109218)

• β-Mercaptoethanol (Sigma Aldrich, cat. no. M6250-500ML)

**[CAUTION!]** β-Mercaptoethanol is volatile with a pungent odor. Make sure that wear a mask and operate in the fume hood during experiments. Dispose should be in accordance with local regulations.

• Copper grids (200 mesh) with coated carbon support film (Beijing Zhongjingkeyi Technology, cat. no. BZ110205b)

• 0.22-μm centrifugal filter (Millipore, cat. no. SLGP033NS)

• Bis-Tris gel (GenScript, cat. no. M00656)

• Q column (GE Healthcare, cat. no. 17-5156-01)

• Superdex 75 (GE Healthcare, cat. no. 28-9893-33)

• 96-well plate (Corning, cat. no. 3590)

• 384-well optical plate (Thermo Scientific, cat. no. 142761)

• Dialysis Clip (Thermo Scientific, cat. no. 87733)

• Filter paper (Jiaojie, GB/T1914-2007)

• Mica sheet (Zhaojing)

• Centrifugal filters 3K (Millipore, cat. no. UFC900396)

• 1.5-mL microtube (Axygen, cat. no. MCT-150-C)

• 15-mL Centrifuge tube (YUEYI, cat. no. YB0019-15)

• 50-mL Centrifuge tube (YUEYI, cat. no. YB-50D)

### Reagent setup

• 2× YT medium. Dissolve 16 g Tryptone, 10 g Yeast extract and 5 g NaCl with deionized water. Bring the volume to 1 L and perform high temperature sterilization. This medium can be stored at room temperature (RT).

• 1 mol/L Tris (pH 8.0) stock. Dissolve 121.14 g Tris in 900 mL deionized water and stir until most of it is dissolved. Adjust the pH to 8.0 with HCl. Bring the volume to 1 L and filter-sterilize the solution. This solution can be stored at RT.

• 3 mol/L KCl stock. Dissolve 223.65 g KCl in 900 mL deionized water and stir until most of it is dissolved. Bring the volume to 1 L and filter-sterilize the solution. This solution can be stored at RT.

• 4 mol/L NaCl stock. Dissolve 233.76 g NaCl in 900 mL deionized water and stir until most of it is dissolved. Bring the volume to 1 L and filter-sterilize the solution. This solution can be stored at RT.

• 1 mol/L Tris (pH 7.5) stock. Dissolve 121.14 g Tris in 900 mL deionized water and stir until most of it is dissolved. Adjust the pH to 7.5 with HCl. Bring the volume to 1 L and filter-sterilize the solution. This solution can be stored at RT.

• 500 mmol/LM EDTA Stock. Dissolve 186.12 g EDTA in 900 mL deionized water and stir until most of it is dissolved. Bring the volume to 1 L and filter-sterilize the solution. This solution can be stored at RT.

• Bacteria lysis buffer. 100 mmol/L Tris (pH 8.0), 1 mmol/L EDTA，1 mmol/L PMSF.

• Bacteria storage buffer. 100 mmol/L Tris (pH 8.0), 1 mmol/L EDTA.

• Buffer A. 25 mmol/L Tris (pH 8.0). This solution can be stored at RT.

**[CAUTION!]** Before each use, ultrasonic debubble and filtration are a must.

• Buffer B. 25 mmol/L Tris (pH 8.0), 1 mol/L NaCl. This solution can be stored at RT.

**[CAUTION!]** Before each use, ultrasonic debubble and filtration are a must.

• Buffer for size exclusion chromatography. 50 mmol/L Tris (pH 7.5), 150 mmol/L KCl.

**[CAUTION!]** Ultrasonic debubble and filtration must be performed before each use.

• Fibril growth and ThT buffer. 50 mmol/L Tris (pH 7.5), 150 mmol/L KCl, 0.05% NaN_3_.

• Loading buffer (5×). Dissolve 5.0 g SDS in 8.33 mL 1.5 mol/L Tris (pH 6.8). Then add 25 mL glycerol, avoiding air bubbles, and bring the volumn to 45 mL with deionized water. After complete dissolution, add 250 mg bromophenol blue and bring the volumn to 50 mL with β-mercaptoethanol.

**[CAUTION!]** β-Mercaptoethanol is volatile with a pungent odor. Make sure that wear a mask and operate in the fume hood during experiments. Dispose should be in accordance with local regulations.

• PMSF (0.2 mol/L). Dissolve 0.70 g PMSF in 16 mL isopropanol. After ultrasonic dissolution, bring the volumn to 20 mL. 1 mL aliquots should be protected from light and stored at –20 °C. PMSF can be stored for one year.

**[CAUTION!]** PMSF is acutely toxic. Wear a mask and handle with protective gloves.

• Insoluble buffer. 50 mmol/L Tris, pH 8.0, 150 mmol/L NaCl, 1% Triton X-100, 2% SDS.

**[CAUTION!]** SDS is harmful. Handle with protective gloves.

• IPTG (1mol/L). Dissolve 23.83 g IPTG in 90 mL deionized water. After complete dissolution, bring the volumn to 100 mL. Aliquots should be stored at –20 °C.

• SDS-PAGE running buffer (1×). Add 25 mmol/L Tris, 192 mmol/L glycine and 0.1% (*w*/*v*) SDS; bring it to the final volume with water. This buffer can be stored indefinitely at RT.

• ThT solution (1 mmol/L). Dissolve 3.19 mg ThT in 9 mL deionized water. After completely dissolved, bring the volumn to 10 mL. Aliquots should be stored at –4 °C.

## EQUIPMENTS

• Pipettes: 25 mL, 10 mL, 5 mL, 1mL

• Syringe: 2 mL, 10 mL, 20 mL, 50 mL

• Pipettor (Eppendorf, Easypet 3)

• Centrifuge tube: 1.5 mL, 5 mL, 15 mL, 50 mL

• −80 °C freezer (Thermo Fisher Scientific)

• Probe tip sonicator (Xinyi, JY92-IIN)

• Ultrasonic cleaner (Kunshan Shumei, KQ5200E)

• Benchtop centrifuge for 1.5-mL tubes (up to 15,000 *g*)

• Thermomixer with block for 1.5-mL tubes (Eppendorf)

• Transmission electron microscope (FEI, Tecnai, 120KV TWIN)

• Glow discharge cleaning system (Ted Pella, PELCO easiGlow)

• Nanodrop 2000c (Thermo Fisher Scientific)

• Akta Pure (Union, UI 1500)

• Cell cracker (Union, UH-06)

• pH meter (Sartorius, PB-10)

• Protein staining apparatus (Genescript, eStain LG)

• Benchtop refrigerated centrifuge (Beckman Coulter, Microfuge 11)

• Microprocessor-controlled mini benchtop centrifuge (Beckman Coulter, Microfuge 16)

• High-speed centrifuge (Beckman Coulter, Avanti j-26S XP)

• Silicon tip on nitride lever (Bruker, SNL-10)

• Double coated adhesive tape (Minnesota Mining and Manufacturing Co.)

• Double-sided tissue tape (Maped)

• Fluoroskan Ascent microplate reader (Thermo Scientific, FLUOstar Omega)

• Circular Dichroism Spectrometer (Applied Photophysics, Chirascan VX)

• Multimode 8 scanning probe microscope (Bruker, Multimode Scan)

• Multimode Adapter (Bruker, NanoScope V)

• Benchtop incubator shaker (New Brunswick Scientific, Innova 40)

• Electrophoresis tank (Bio-rad, Mini-Protean Tetra System)

• Magnetic Stirrer (DLAB, MS-H-PRO)

• Vortexer (Shanghai Qite, QT-2A)

• Oscillating incubator (Infors, Multitron)

## Conflict of interest

Houfang Long, Shuyi Zeng, Yunpeng Sun and Cong Liu declare that they have no conflict of interest.
